# Mediators of the longitudinal relationship between childhood adversity and late adolescent psychopathology

**DOI:** 10.1017/S0033291721000477

**Published:** 2022-11

**Authors:** Colm Healy, Aisling Eaton, Isabel Cotter, Ellen Carter, Niamh Dhondt, Mary Cannon

**Affiliations:** 1 Department of Psychiatry, Royal College of Surgeons in Ireland, Dublin 2, Ireland; 2 Department of Psychology, Dublin City University, Dublin 9, Ireland; 3 School of Medicine, University of Dublin Trinity, Dublin 2, Ireland; 4 Department of Psychiatry, Beaumont Hospital, Dublin 9, Ireland

**Keywords:** Childhood adversity, parent-child conflict, psychopathology, self-concept and physical activity

## Abstract

**Background:**

Childhood adversity (CA) is commonly associated with an increased risk of subsequent psychopathology. It is important to identify potential mediators of this relationship which can allow for the development of interventions. In a large population-based cohort study we investigated the relationship between CA and late adolescent psychopathology and early adolescent candidate mediators of this relationship.

**Methods:**

We used data from three waves (*n* = 6039) of Cohort 98′ of the Growing up in Ireland Study (age 9, 13 and 17). We used doubly robust counterfactual analyses to investigate the relationship between CA (reported at age-9) with psychopathology (internalizing and externalizing problems), measured using the Strengths and Difficulties Questionnaire at age-17. Counterfactual and traditional mediation was used to investigate the mediating effects of the parent-child relationship, peer relations, self-concept, computer usage and physical activity.

**Results:**

CA was associated with an increased risk of internalizing and externalizing problems at age-17. Parent-child conflict mediated 35 and 42% of the relationship between CA and late adolescent externalizing problems and internalizing problems, respectively. Self-concept and physical activity mediated an additional proportion of the relationship between CA and internalizing problems. These results were robust to unmeasured confounding.

**Conclusions:**

Parent-child conflict explains more than a third of the relationship between CA and later psychopathology. Self-concept and physical activity explain the additional proportion of the relationship between CA and internalizing problems. This suggests that these factors may be good targets for intervention in young people who have experienced CA to prevent subsequent psychopathology.

## Introduction

Childhood adversity (CA) is common (Zhang et al., 2020), with estimates from international studies showing that one-third of children report 1–2 adverse experiences, and approximately one-quarter report 3 or more (Albott, Forbes, & Anker, 2018). A relationship between CA and subsequent development of mental health difficulties has been observed among epidemiological and clinical samples (Aafjes–van Doorn, Kamsteeg, & Silberschatz, 2020). CA has been associated with poorer physical and mental health in adulthood (Green et al., 2010; Hughes et al., 2017) and has been implicated in the development of many mental disorders, including depression, schizophrenia, psychotic symptoms and personality disorder (Belbasis et al., 2018; Coughlan & Cannon, 2017; Lenze, Xiong, & Sheline, 2008; and Pietrek, Elbert, Weierstall, Müller, & Rockstroh, 2013). As such, CA can be considered a transdiagnostic risk factor for psychopathology (Albott et al., 2018).

While it can be difficult to prevent CA from occurring, research has identified buffering factors in the relationship between CA and development of psychopathology, which span social, emotional and neurobiological domains (McLaughlin & Lambert, 2017). Cicchetti (2016) advised that future investigations should focus on examining the role of mediators ‘in order to better understand the systems that underlie the developmental consequences of maltreatment.’ Mediation analysis can be used to identify the mechanisms by which two variables might be related and therefore can highlight potential intervention targets in those who have been exposed to a major risk factor for an unwanted outcome (Lapointe-Shaw et al., 2018). In this circumstance, mediators would seek to explain why CA is linked with mental disorders.

Several mediators have been identified as underlying the relationship between CA and subsequent psychiatric outcomes. These include the mediating effect of familial attachment style on depressive symptoms (Hankin, 2005), parent-child conflict and peer relationship on externalizing problems (Dmitrieva, Chen, Greenberger, & Gil-Rivas, 2004; Kim & Cicchetti, 2010). Self-concept, the sum of an individual's beliefs and knowledge about his/her personal attributes and qualities (Healy et al., 2019), has been shown to partially mediate the relationship between negative life events with depressive symptoms, suicide thoughts and behavior (Wong, Dirghangi, & Hart, 2019). Physical activity has been associated with CA and it partially accounts for a proportion of the relationship between CA with symptoms of PTSD and depression (Chen et al., 2020; Fasciano et al., 2020). Finally, maladaptive internet use has been associated with both CA and psychopathology (Carli et al., 2013; Yates, Gregor, & Haviland, 2012) and the potential for maladaptive internet use to mediate the relationship between CA and psychopathology warrants further investigation. Preacher and Hayes (2008) suggested that simultaneously testing several mediators minimizes the risk of excluding relevant factors and identifying a single process as the only mediator.

A previous analysis from our group (Dhondt, Healy, Clarke, & Cannon, 2019) found that parent-child conflict in childhood mediated the relationship between CA and psychopathology at the beginning of adolescence. As a phase of development, adolescence comprises rapid biological and psychosocial change (Branje, 2018). It is possible that mediators are time-period sensitive and what is effective for reducing the risk of early-adolescent psychopathology may differ from what is effective for reducing the risk of late-adolescent psychopathology. This current study examines the potential mediating effects of early adolescent candidate mediators on psychopathology in late adolescence. This study had two aims. Firstly, we aimed to investigate the relationship between CA and internalizing and externalizing problems at age 17. Secondly, we aimed to investigate a range of potential early adolescent mediators for the relationship between CA and psychopathology in late adolescence. These potential mediators comprised parent-child conflict, parent-child positive, self-concept, peer trust, peer alienation, amount of computer use and physical activity.

## Method

### Participants

The current sample consisted of the child cohort (Cohort 98′) of the ‘Growing Up in Ireland’ (GUI) study – a national, longitudinal study that examined contributing factors to the development of children in modern Ireland (Murphy, Williams, Murray, & Smyth, 2019). The sample design of the child cohort of GUI involved a two-stage recruitment process of 9-year-olds (Greene et al., 2010). This involved the random selection of 910 national schools, as primary sampling units, and then children within the age range were selected within each school. Recruited students were selected to accurately reflect the Irish population of 9-year-olds with 8658 children and their families participating.

The second wave of the study (age 13) included the full target sample of the 8658 children who completed the first wave (Thornton, Williams, McCrory, Murray, & Quail, 2016). In total, 7423 families agreed to participate (87.7% retention rate). At the third wave of the study (age 17/18) 6216 families, of the 8568 that participated at wave one participated (74% retention rate; Murphy et al., 2019). Because the present study is examining data at 9, 13 and 17–18-years old, only those who participated in all three waves of the study were deemed eligible for the current study sample (*n* = 6039, 70% retention rate of the original sample; Murphy et al., 2019). To ensure the data were nationally representative it was reweighted to account for sampling bias and attrition. Thus, the final reweighted sample is representative of children who were residing in Ireland at 9 years of age in 2006, and who continued to live in Ireland at 17/18 years of age in 2016.

#### Ethical considerations

The GUI received ethical approval from the Health Research Board's research ethics committee in Ireland. Participants gave assent/consent at each wave of the study while participant's primary caregivers also gave consent.

### Exposures

#### Childhood adversity

Primary caregivers of 9-year-olds were asked about the participating child's exposure to a set of 14 adverse life events. These adverse experiences were the death of a parent, the death of a close family member, the death of a close friend, a parent in prison, drug-taking or alcoholism in the immediate family, mental disorder in the immediate family, a stay in a foster home or residential care, serious illness or injury, serious illness or injury of a family member, divorce or separation of parents, conflict between parents, moving house, moving country or another unspecified, disturbing event. The questionnaire did not include any measure of physical abuse, sexual abuse or neglect of the participating child.

In line with the methods of Dhondt et al. (2019) we defined CA as experiencing three or more adverse life events or experiencing at least one severe life events. Briefly, we sought to create a measure that acknowledges the potential for cumulative adverse events as well as acknowledge that some adverse events are more severe than others. The severe events were considered to be the death of a parent, death of a close friend, parent in prison, drug-taking or alcoholism in the immediate family, mental disorder in the immediate family, serious illness or injury and a stay in a foster home or residential care. The events severity were decided by a consensus meeting.

Due to the subjective nature of our adversity variable, we conducted two online supplementary analyses using alternative measures of CA. In the first online supplementary analysis, we used a cumulative CA measure. Cumulative CA was defined as the sum of the endorsement of each adverse life event. In the second online supplementary analysis, we derived a latent CA measure using item-response-theory (see online supplementary materials for details and analyses).

### Outcomes

#### Late adolescent psychopathology

The Strengths and Difficulties Questionnaire (SDQ; Goodman, 1997) was administered to parents and guardians of the participating young person at 17–18-years-old. This is a 25 item questionnaire measuring prosocial behavior and four psychopathology sub-scales; emotional symptoms, conduct problems, hyperactivity or inattention, peer problems. Primary caregivers were required to respond to each item (i.e. not true, somewhat true, certainly true; Murphy et al., 2019).

Sum scores for internalizing and externalizing problems were used separately. Threshold scores for internalizing and externalizing behaviors were predefined, according to the measure definition. The threshold score for internalizing problems was seven or more, while the score for externalizing problems was nine or more.

### Mediators

Potential mediators were chosen based on the available evidence in the literature.

Data on all mediators were collected at age 13 from either the primary caregiver or the participating child.

- *Parent-Child Relationship*. The Child-Parent Relationship Scale (Pianta, Nimetz, & Bennett, 1997) was used to assess the parent or guardian's relationship with the participating child. Within the GUI, two subscales were used; the parent-child conflict subscale and the parent-child positive subscale. These scales measured the positive and negative aspects of the relationship and the primary caregiver rated each statement on a five-point scale. Both subscales were measured as continuous variables.
- *Self-Concept*. The participating child's self-concept was measured using a 60-item Piers-Harris II Children's Self-Concept Scale (Piers, Herzberg, & Harris, 2002). This measure includes sub-scales assessing behavioral adjustment, intellectual and school status, physical appearance and attributes, freedom from anxiety, popularity and happiness and satisfaction. The total score of these subscales was used in the analyses and measured as a continuous variable.
- *Peer Relationships and Attachment*. The Inventory of Parent and Peer Attachment (IPPA; Armsden and Greenberg, 1987) was used to measure the participating child's positive and negative perceptions of the affective and cognitive aspects of their relationships with peers (Thornton et al., 2016). Participants in the GUI were asked to complete two sub-scales of the IPPA: Peer Trust and Peer Alienation. The IPPA uses a five-point Likert scale for response format. Both sub-scales were measured as continuous variables.
- *Computer Use*. The participating child's computer use was measured using a continuous variable examining the amount of time the child dedicated to computer-use on a given weekday. This was a self-report measure responded to by the child. The question asked was ‘On a normal weekday, during term-time, how much time did the child spend on the computer, in hours and minutes, excluding computer-use in school?’ Responses were recorded in 30 min blocks ranging from 0 min to greater than 6 h.
- *Physical Activity*. The study child's level of physical activity was assessed using an ordinal variable assessing the amount of hard physical activity the child completed in the 14 days prior to the questionnaire. This was a self-report, responded to by the child. The question asked how many times in the prior 14 days did the child complete at least 20 min of hard exercise (i.e. enough to make their breathing fast and make their heart beat faster). This was a five-point measure, with answers ranging from no days out of the 14 to 9 or more days.

### Confounders

Confounding variables include gender, socioeconomic status (as measured by income quintile and the primary care giver's highest level of education), nationality (Irish or non-Irish) and urbanicity (living in a rural or urban setting).

### Statistical analysis

Descriptive statistics and logistic regression were used to investigate differences between those with and without CA. The relationship between CA and late adolescent psychopathology was investigated using doubly robust counterfactual analysis (inverse probability weighting with regression adjustment). We report the potential outcome means, risk difference [average treatment effect (ATE) and the average treatment effect in the treated (ATET)], risk ratio, the population attributable fraction. Potential outcomes are defined as the set of possible outcomes that an individual would have obtained had they been experienced each level of the exposure. In this study, it refers to what would have been the risk of psychopathology in a child who had not experienced adversity, had they experienced adversity (the counterfactual outcome) and vice-versa. Potential outcome means are the averaged potential outcomes in the exposed and unexposed groups after calculating all counterfactual outcomes.

**Fig. 1.**
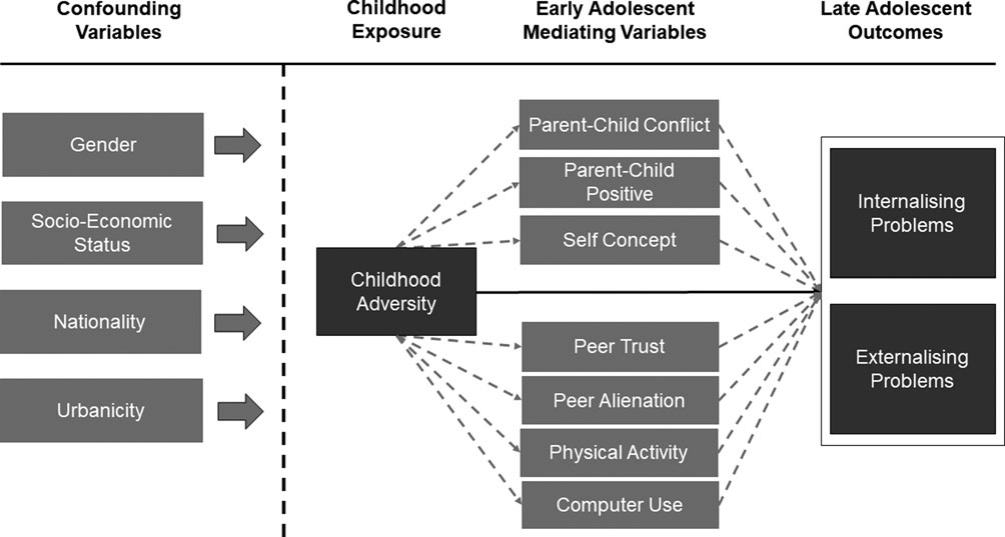
A visual schematic of the relationship between confounders, exposure, mediators and outcomes.

In line with Baron and Kenny (1986) we investigated the relationship between the exposure and the mediator and the mediator and the outcome using linear and logistic regression. Univariate counterfactual mediation was conducted using the *medeff* command and sensitivity bias analysis was conducted using the *medsens* command in Stata 15 (Hicks & Tingley, 2012). Multivariate mediation was conducted using traditional mediation with the KHB path decomposition command (Kohler, Karlson, & Holm, 2011). A visual schematic of the proposed model is displayed in Fig. 1.

Online Supplementary Analysis (see supplementary materials): In the first online supplementary analysis, we examine the individual and cumulative risk of late adolescent psychopathology using logistic regression. In the second online supplementary analysis, we derived a latent measure of CA using two-parameter model item-response theory. Logistic regression was used to examine the relationship between this latent adversity measure and late adolescent psychopathology. In both online supplementary investigation, mediation analysis was conducted using the same procedure as a report in the main text (the medeff command was used for the univariate mediation and KHB path decomposition was used for multi-variate mediation). All analyses were conducted using Stata 15 (StataCorp, 2017).

## Results

### Prevalence and demographics of CA

In total 78.7% of the sample had experienced at least one adverse life event by age 9. We found that 28% (*n* = 1713) of participants met the definition of CA at 9-years-old of whom 20% (*n* = 1207) participants experienced three or more adverse life events, while 18% (*n* = 1067) had experienced at least one of the severe adverse life events. The demographics of those who did and did not report CA are reported in Table 1. Those who experienced CA were more likely to be born outside of Ireland and come from a lower socio-economic background than their peers (Table 1).

**Table 1.** Demographic information of those who reported child adversity and those who did not

Characteristic	Overall	Controls	Child adversity	Odds ratio
Gender (% female)	48.96	47.93	51.56	1.16 (0.98–1.36)
Child nationality (% not born in Ireland)	10.47	7.25	18.61	**2.93** (2.3–3.72)
PCG nationality (% not born in Ireland)	14.88	13.16	19.22	**1.57** (1.27–1.95)
PCG's highest education				
None/Primary	5.62	4.54	8.34	**2.23** (1.46–3.4)
Junior certificate or equivalent	23.54	22.35	26.52	**1.44** (1.15–1.8)
Leaving certificate or equivalent	37.07	39.01	32.18	Reference
Non-degree	16.46	16.39	16.63	**1.23** (1.00–1.52)
Primary degree	11.18	11.34	10.78	1.15 (0.9–1.47)
Post-graduate	6.13	6.36	5.55	1.06 (0.77–1.44)
Annual family income quint (%)				
Lowest	18.17	16.74	21.74	**1.33** (1.00–1.76)
Second	20.98	19.32	25.12	**1.33** (1.03–1.71)
Third	20.44	20.56	20.14	Reference
Fourth	20.26	21.55	17.02	0.81 (0.63–1.04)
Highest	20.15	21.82	15.98	**0.75** (0.58–0.96)
Region (% urban)	43.98	43.26	45.78	1.11 (0.94–1.30)

*Note*: Emboldened responses denote *p* < 0.05.

#### CA and adolescent psychopathology

*Internalizing Problems**.*** In late adolescence, 15.2% of young people had internalizing problems. Of the total, 35% of those with internalizing problems had a history of CA. Using a doubly robust method we estimated the potential outcome means (see Table 2). The results suggest that child adversity had a population attributable fraction of 7% (risk ratio: 1.29, 95%ile CI1.07–1.55). A similar risk difference was seen when the analysis was restricted to only those who had experienced child adversity (ATET: 3.88%, 95% CI 0.44–7.31).

**Table 2.** Statistical metrics for the relationship between childhood adversity and late adolescent psychopathology

	Internalizing problems	Externalizing problems
Potential outcome means		
Child adversity	0.18 (0.16–0.21)	0.11 (0.09–0.14)
No child adversity	0.14 (0.13–0.16)	0.06 (0.05–0.08)
Risk difference (ATE – marginal effect)	**0.04** (0.01–0.07)	**0.05** (0.02–0.08)
Risk ratio	**1.29** (1.07–1.55)	**1.78** (1.34–2.37)
Population attributable fraction	7.26%	17.79%

*Note*: ATE, average treatment effect. Parentheses indicate the 95%ile confidence intervals. Emboldened metrics denote significant differences (*p* < 0.05).

*Externalizing Problems**.*** In late adolescence, 7.5% of young people had externalizing problems. Of those with externalizing problems, 42% had a history of CA. Child adversity had a population attributable fraction of 18% (risk ratio:1.78, 95%ile CI 1.34–2.37). A similar risk difference was observed when the analysis was restricted to only the children exposed to adversity (ATET:4.80%, 95% CI 1.79–7.81).

#### Mediation analysis

CA *and Early Adolescent Mediators**.*** CA was associated with increased early adolescent parent-child conflict at 13-years-old (Table 3). CA was associated with lower positive parent-child relations, self-concept and physical activity (Table 3). CA was not significantly associated with peer trust, peer alienation or computer use thus were not considered mediators in subsequent analyses (Table 3).

**Table 3.** The association between candidate mediators, exposures and outcomes

	Candidate mediators						
	Parent-child conflict	Parent-Child positive	Self-concept	Peer trust	Peer alienation	Physical activity	Computer use
Exposure (age 9)^a^							
Childhood adversity	**1.46** (0.87–2.05)	**−0.29** (−0.57 to −0.02)	**−1.45** (−2.23 to −0.67)	−0.29 (−0.95 to –0.37)	0.28 (−0.11 to –0.66)	**−0.15** (−0.25 to −0.05)	0.19 (−0.60 to –0.44)
Outcomes (age 17//18)^b^							
Internalizing problems	**1.09** (1.07–1.10)	**0.94** (0.91–0.96)	**0.95** (0.93–0.96)	**0.96** (0.95–0.97)	**1.06** (1.03–1.08)	**0.77** (0.69–0.84)	**1.07** (1.04–1.11)
Externalizing problems	**1.16** (1.13–1.18)	**0.91** (0.88–0.94)	**0.97** (0.95–0.98)	**0.97** (0.95–0.99)	**1.04** (1.00–1.08)	**0.88** (0.78–0.99)	**1.07** (1.01–1.12)

*Note*: Emboldened metrics denote significant differences (*p* < 0.05).

a The investigation between exposures and the mediators was investigated using linear modeling.

b The relationship between mediators and exposure/outcome were investigated using binary modeling. All analyses are weighted.

*Early Adolescent Mediators and Late Adolescent Outcomes**.*** Parent-child conflict, Peer alienation and higher computer usage in early adolescence were significantly associated with an increase in internalizing and externalizing problems in late adolescence (Table 3). Lower parent-child positive relationship, self-concept, peer trust and physical activity were associated with an increase in internalizing and externalizing problems in late adolescence.

#### Counter-factual mediation

*Internalizing Problems**.*** In a univariate counter-factual mediation analysis, all remaining candidate variables mediated a proportion of the relationship between child adversity and internalizing problems in late adolescence (Table 4). The percentage mediation varied across the mediators with parent-child conflict accounting for the largest percentage of the relationship. The direct effect between CA and late adolescent internalizing problems was only retained when a positive parent-child relationship was the candidate mediator. When self-concept, parent-child conflict and physical activity were the candidate mediators there was no significant direct association between child adversity and late adolescent internalizing problems. To account for potentially competing pathways, a secondary analysis (non-counterfactual multivariate path-decomposition analysis) was conducted. This revealed that parent-child conflict, self-concept and physical activity combined accounted for over 75% of the relationship between CA and late adolescent internalizing problems (total indirect odds ratio:1.20, 95%ile CI 1.12–1.29) and each variable uniquely mediated a proportion of this pathway (see Table 4). Parent-child conflict mediated the largest proportion of the relationship.

**Table 4.** Counterfactual risk differences of the total, direct and indirect relationship between childhood adversity and late adolescent psychopathology

Mediator variables	Single variable counterfactual mediation^a^				Multivariate non-linear probability path-decomposition	
	Total effect *RD*	Average mediation *RD*	Average direct effect *RD*	Percentage of average mediated effect	Average mediation *OR*	Percentage of the average mediation
Childhood adversity and adolescent internalizing problems						
Parent-child conflict	**3.53** (3.05–7.13)	**1.50** (0.89–2.18)	2.03 (−1.17 to –5.35)	**42.36%**	**1.10** (1.06–1.16)	**42**.**27**
Parent-child positive	**3.91** (0.64–7.22)	**0.24** (0.01–0.52)	**3.67** (0.47–6.98)	**6.27%**	1.00 (0.99–1.01)	1.49
Self-concept	**3.36** (0.07–6.63)	**1.00** (0.47–1.61)	2.36 (−0.82 to –5.63)	**29.38%**	**1.07** (1.03–1.11)	**27**.**22**
Physical activity	**3.58** (0.35–6.89)	**0.49** (0.16–0.90)	3.09 (−0.11 to –6.40)	**13.58%**	**1.02** (1.00–1.04)	**9**.**40**
Childhood adversity and adolescent externalizing problems						
Parent-child conflict	**4.24** (1.67–7.31)	**1.47** (0.84–2.12)	**2.77** (0.06–5.68)	**34.97%**	**1.24** (1.13–1.34)	**31**.**12**
Parent-child positive	**4.90** (2.09–7.82)	**0.21** (0.01–0.44)	**4.69** (1.94–7.65)	**4.37%**	0.99 (0.97–1.01)	1.58
Self-concept	**5.11** (2.23–8.06)	**0.33** (0.12–0.60)	**4.78** (1.98–7.79)	**6.50%**	**1.03** (1.00–1.06)	**3**.**76**
Physical activity	**5.15** (2.29–8.16)	0.13 (−0.01 to –0.32)	**5.02** (2.19–8.05)	2.54%	–	–

*Note*: RD, risk difference; OR, odds ratio. Emboldened metrics denote significant differences (*p* < 0.05). All results were adjusted for gender, income, PCG highest level of education, urbanicity, nationality.^a^: Only variables significantly associated with the exposure and outcome were examined as potential mediators.

*Externalising Problems**.*** Univariate counter factual mediation demonstrated that parent-child conflict, parent-child positive and self-concept each mediated a significant proportion of the relationship between CA and externalizing problems (see Table 4). Physical activity did not significantly mediate this pathway. A significant direct effect was still observed in all analysis. To account for competing pathways, multivariate mediation was conducted. In the multivariate path decomposition parent-child conflict mediated roughly a third of the relationship between CA and externalizing problems. Additionally, self-concept mediated a small proportion of this relationship (<5%).

#### Sensitivity bias

In an attempt to account for unmeasured confounding, we conducted sensitivity bias analysis (see online Supplementary Table S1). The results revealed that a confounder would have to explain between 30% and 50% of the mediator-outcome variance to remove the effects of parent-child conflict. Similarly, 30% of the mediator-outcome variance would have to be explained to remove the effect of self-concept and physical activity in the case of internalizing problems.

#### Supplementary analyses

We carried out two supplementary analyses using different definitions of CA; a cumulative CA measure and a latent CA measure derived from item-response theory. Both measures were significantly associated with late adolescent psychopathology (see online Supplementary Table S2 and Supplementary Materials page 6). Both mediation analyses demonstrated the same pattern of results as was seen in the main analyses; parent-child conflict significantly mediated roughly a third of the relationship between CA and late adolescent psychopathology. Self-concept and physical activity specifically mediated an additional proportion of the relationship between CA and internalizing problems (see online Supplementary Tables S4 and S7).

## Discussion

From this longitudinal, nationally representative sample, we found that CA (as measured as exposure to three or more minor stressors or one major stressor by age 9) was longitudinally associated with internalizing and externalizing problems in late adolescence. We found that parent-child conflict at age 13 mediated a large proportion of the relationship between CA and later psychopathology. In addition, self-concept and physical activity specifically mediated the relationship between CA and internalizing problems. Additionally, online supplementary analyses using two different definitions of CA (cumulative childhood stressors and a latent CA variable) reinforced these findings.

These findings expand on our previous work investigating mediators between CA and early adolescent psychopathology (Dhondt et al., 2019) by suggesting that, in the context of CA, early adolescent interventions targeting parent-child conflict, self-concept and physical activity can reduce the risk of late adolescent internalizing and externalizing problems.

The most powerful single mediator found in these analyses was parent-child conflict. Parent-child conflict has previously been shown to mediate the relationship between adverse life events and externalizing problems (Dmitrieva et al., 2004). These results align with the protective effects that secure attachment may have in the context of adverse life events (Nowalis et al., 2020). For example, CA may strain the relationship between primary caregiver and child. A secure attachment with the primary caregiver may off-set the degree of parent-child conflict induced by the adversity. This, in turn, may reduce the risk of subsequent psychopathology.

Interventions in this area have mainly focused on parental education and home visits. Positive parenting practices have been shown to protect against adversity in young children and interventions can be designed to promote these practices at home for the children of families suffering high levels of adversity. In terms of population – wide interventions and children with lower risk, family and cognitive therapy, based around solving and communication (Barkley, Fischer, Smallish, & Fletcher, 2002) have been shown to have moderate effects but are intensive, making universal or selected implementation challenging. Evidence-based policies such as home visits and parent resources may be a more feasible solution (Yamaoka & Bard, 2019). However, causes of parent-child conflict vary widely following CA and as such, a personalized form of intervention may be required that takes into account the multiple contributors to the problem.

Self-concept was found to mediate the relationship between CA and internalizing problems in this study. This is in line with previous research which demonstrated that, in young people, self-concept mediates the relationship between poly-victimization and psychological distress (Turner, Shattuck, Finkelhor, & Hamby, 2017). Several interventions have been to be effective for improving self-concept (O'Mara, Marsh, Craven, & Debus, 2006). For example, a universal school-based programs have also been shown to improve self-concept through mental health literacy and dialectical behavior therapy skills programme (Katz, Mercer, & Skinner, 2020). However, the downstream effects of such an intervention on psychopathology have yet to be examined. Additionally, it is unclear whether these programs are effective in young people who have experienced CA or trauma. Additional research specifically examining the effects of self-concept interventions in those who have experienced adversity would be beneficial.

Previous research has shown that physical activity can have a considerable influence on mental health burden (McMahon et al., 2017). Physical activity interventions can be effective in reducing psychopathology (Spruit, Assink, van Vugt, van der Put, & Stams, 2016a), while CA has been shown to increase the risk of physical inactivity in adulthood (Bellis et al., 2014). Interestingly, in our study physical exercise only mediated the relationship between CA and internalizing problems. The evidence for the association between physical activity and externalizing problems is limited (Chamberlain, 2013). A recent meta-analysis found no overall association between sports participation and juvenile delinquency (Spruit, Van Vugt, Van der Put, & Van der Stouwe, 2016b). It is not known to what extent the positive effect of physical activity on mental health is due to the exercise itself or the social benefit. Exercise has also been found to positively affect self-concept, stress-management and social skills (Alfermann & Stoll, 2000). Thus, it may act on multiple factors be they biological, psychological or social.

Peer relationships and computer use did not mediate the relationship between CA and adolescent psychopathology. Within our investigation, CA was not associated with peer trust or peer alienation suggesting that adversity does not alter the relationship between the child and their peers. However, lower peer trust and high peer alienation were found to be risk factors for late adolescent psychopathology. Previous results have demonstrated that poor peer relations are associated with adverse outcomes in adolescence such as internalizing symptoms and self-harm (Reijntjes, Kamphuis, Prinzie, & Telch, 2010). Peer relationship has previously been shown to mediate the pathway between physical abuse and neglect with subsequent psychopathology (Ban & Oh, 2016). However, within our study, the adversity measure did not include data pertaining to trauma or neglect. It is possible that these mitigating effects of peer relationship are only apparent in more severe forms of trauma.

We found an association between time spent on computers and adolescent psychopathology, though as with peer relationships, it was not associated with CA. Greater screen-time has been associated with an increased risk of adolescent psychopathology (Twenge & Campbell, 2018). However, the duration of computer use alone may not accurately capture maladaptive/harmful use (Carras & Kardefelt-Winther, 2018) and it is possible that certain computer use features may be specifically associated with CA and subsequent psychopathology. Unfortunately, our metric is unable to distinguish between adaptive and maladaptive use beyond the duration of computer use.

### Strengths and limitations

The size and representative nature of the sample used and the validity of the outcome measures strengthen the results and conclusions. We used counterfactual analysis to estimate the effects of CA on psychopathology and in our mediation analyses. We used sensitive analysis to assess the robustness of our findings to unmeasured confounding. Additionally, we used longitudinal data with the exposure, mediators and outcomes all measured at separate time points.

One limitation is that we were reliant on parent-reported measures for outcome. There is some evidence that child-reported measures of psychopathology differ from parent-reported ones, especially when the child is in adolescence (Van Roy, Groholt, Heyerdahl, & Clench-Aas, 2010). This may lead to an underestimation of the proportion of internalizing and externalizing problems (Van Roy et al., 2010). Another limitation is that we do not have data on severe traumatic experiences such as sexual, emotional or physical abuse in the GUI study. However, both severe and moderate stressful life events appear to cluster in time and their absence from the measurement does not mean they exercised no effect on the results (Costello, Erklani, Fairbank, & Angol, 2002).

It is possible that different types of adverse life events will have a different impact on the risk of internalizing and externalizing problems (see online Supplementary Table S3), and in certain circumstances may be differentially mediated. For example, altering the parent-child relationship may be more effective at reducing the risk of subsequent psychopathology in the context of an unstable family environment, whereas altering self-concept may be more effective in the context of the child experiencing a serious illness or injury. Most of our sample had experienced at least one type of adverse event (~79%) with 44% has experienced two or more adverse events (see online Supplementary Table S2). Thus, for clarity, while our three measures of CA provide consistent evidence for the mediators of the CA and psychopathology relationship, they do not speak to stressor specific association with psychopathology. Conducting multiple stressor specific investigations would likely increase the risk of false-positive observations and it does not provide a broad framework for mediators of CA-psychopathology relationship. Further research is required to investigate if the association between specific stressors and psychopathology are differentially mediated.

Finally, our results are based on observational data. Mediation analysis has strong causal assumptions that observation studies typically violate. We attempted to account for this with the counterfactual mediation. There is also a possibility of residual confounding from unmeasured confounders but our sensitivity analysis suggests that they would have to account for a large proportion of the mediator outcome relationship to invalidate our primary findings. We assessed multiple mediators simultaneously. The cross-sectional nature of our mediators means they probably interact with one another and the causal nature of the within mediator association is unknown.

## Conclusion

Adversity is commonly experienced in childhood and is sometimes an unavoidable consequence of life circumstances. We demonstrate that CA is associated with an increased risk of late adolescent psychopathology. We demonstrate that reducing parent-child conflict in early adolescence may play a key role in mitigating the effect of adversity on the likelihood of developing internalizing and externalizing problems in late adolescence. Our findings suggest that, in the context of adversity, physical activity and self-concept in early adolescence may also be useful targets for intervention for reducing the risk of late adolescent internalizing problems. We advocate for future studies on parent-child conflict, self-concept and physical activity as intervention targets in those who have experienced CA as this may reduce the burden of psychopathology in vulnerable youth (Healy & Cannon, 2020).

## Supplementary material

For supplementary material accompanying this paper visit https://doi.org/10.1017/S0033291721000477. click here to view supplementary material
