# Supplementary material for: Mediators of the longitudinal relationship between childhood adversity and late adolescent psychopathology
Source: Psychol Med. 2021 Mar 3;52(15):3689–97. doi: 10.1017/S0033291721000477 (PMC9772905; doi:10.1017/S0033291721000477)
Supplement: Supplementary file 1 [file S0033291721000477sup001.docx]

**Supplementary materials.**

**Supplementary Materials Sensitivity Bias**

The medsens command in Stata (StataCorp, 2017) was used to derive the percentage to which unmeasured confounding would have to explain in the mediator outcome relationship for our average mediating effect to become zero (no effect).

Supplementary Table 1. Sensitivity bias for the univariate counterfactual analyses.

| **Candidate Mediators** | **Internalizing Problems** | | **Externalizing Problems** | |
| --- | --- | --- | --- | --- |
|  | **Mediation (%)** | **Rho (%) Ye/Me** | **Mediation (%)** | **Rho (%) Ye/Me** |
| **Parent Child Conflict** | 42.4 | 30 | 35.0 | 50 |
| **Parent Child Positive** | 6.3 | 20 | 4.4 | 30 |
| **Self-Concept** | 29.4 | 30 | 6.5 | 30 |
| **Physical Activity** | 13.6 | 30 | 2.5 | 10 |

Note: Rho % Y_E_/M_E_: is the correlation represented as a percentage of the residual variance in both the mediator and the outcome that a confounder must explain for the average mediation effect to become zero (no effect).

**Supplementary analyses**

To bolster the evidence for the mediating effects, we ran two supplementary analysis using two additional approaches to measuring childhood adversity; a cumulative adversity measure; and a measure derived using item response theory. The cumulative adversity variable indicated that exposure to each new stressor increases the risk of psychopathology in adolescence (supplementary analysis one). However this variable will treats all stressors as equally contributing degrees of adversity. Our early life stress items vary widely in severity and this assumption of equality may be inaccurate (see supplementary Table 3). To further strengthen our claims, we conducted a third analysis. This examined the mediators of the relationship between stress and psychopathology, where the adversity variable was calculated using 2-parameter item response theory (2pl-IRT). This latent adversity score accommodates both the cumulative effect of each stressor as well as the variability in stressor severity in a data driven approach (see supplementary analysis two).

**Supplementary analysis one: Cumulative Adversity Measure.**

**Exposure-Outcome Relationship.** We report both in cumulative and individual effects of each stressor on the risk of internalising and externalising problems. Cumulative stress increased the risk of both internalising and externalising problems (see supplementary Table 2). Some, but not all, of the early adverse life events were associated with an increased risk of internalising and externalising problems (see supplementary Table 3). Additionally we demonstrated that, even after you exclude the items that were individually associated with late adolescent psychopathology, there is a significant cumulative effect of stressors on late adolescent psychopathology (see supplementary materials Table 4).

***Exposure-Mediator Relationship.*** Our cumulative adversity measure adversity was significantly associated with parent-child conflict (β =0.66, 95%ileCI: 0.45-0.87), parent-child positive (β = -0.13, 95%ileCI:-0.23 - -0.03), self-concept (β =-0.59, 95%ileCI: -0.86 - -0.31), computer use (β = 0.12, 95%ileCI: 0.03 – 0.21), and physical activity (β =-0.05, 95%ileCI: -0.08 - -0.01). Childhood adversity was not significantly associated with peer trust (β = -0.15, 95%ileCI: -0.37 - -0.07) and peer alienation (β =0.07, 95%ileCI: -0.05-0.20). All mediators were significantly associated with internalising and externalising problems (see Table 2 in the main text).

**Mediation Analysis.** The same statistical methods were was used as in the main text. The results are reported in supplementary table 4 below. In spite of the differences in approaches to calculating our adversity measure between our analysis in the main text (three or more minor stressors and/or one major stressor) and a cumulative stressors count measure, the results are consistent. Parent-childhood conflict accounts for roughly a third of the relationship between our cumulative adversity measure and late adolescent psychopathology (both internalising and externalising problems). Self-concept and physical activity significantly mediated an additional proportion of the relationship between childhood adversity and late adolescent internalising problems which cumulatively accounted for the majority of this relationship.

Supplementary Table 2. The cumulative effect of stressors on psychopathology

| **Number of Stressors** | **%** | **Externalising Problems**  **OR (95%CI)** | **Internalising Problems**  **OR (95%CI)** |
| --- | --- | --- | --- |
| None | 21.3 | - | - |
| One or more | 78.7 | **1.47**  (1.03-2.10) | **1.63**  (1.27-2.08) |
| Two or more | 44.7 | **1.87**  (1.40-2.49) | **1.48**  (1.27-2.08) |
| Three or more | 20.0 | **1.77**  (1.26-2.49) | **1.42**  (1.12-1.80) |
| Four or more | 8.1 | **2.19**  (1.39-3.45) | **1.67**  (1.18-2.37) |
| Five or more | 3.3 | **3.68**  (1.97-6.87) | **2.52**  (1.51-4.21) |
| Cumulative effect | | **1.27**  (1.15-1.41) | **1.17**  (1.09-1.26) |
| Cumulative effect of stressors that do not have an individual effect on outcomes (see supplementary table 2).* | | **1.19**  (1.01-1.40) | **1.13**  (1.02-1.25) |

Note: *: Stressors include =The death of a close family member, moving country, a serious injury or illness of close family members, another unspecified event, the death of a close friend, a stay in foster home or residential care, a parent in prison and a serious injury or illness (participating child). OR: Odds Ratio. Emboldened metrics denote significant differences (p <.05).

Supplementary Table 3. The prevalence of each type of stressor and odds ratios for the relationship with psychopathology.

| **Childhood adversity types** | **%** | **Externalising Problems**  **OR (95%CI)** | **Internalising Problems**  **OR (95%CI)** |
| --- | --- | --- | --- |
| The death of a close family member | 43.2 | 1.03  (0.77-1.39) | 1.11  (0.91-1.36) |
| Moving House | 41.4 | **1.34**  (1.00-1.79) | 1.17  (0.96-1.43) |
| Moving Country | 9.6 | 0.96  (0.57-1.61) | 1.06  (0.75-1.49) |
| Divorce or separation of parents | 14.1 | **2.02**  (1.37-2.99) | **1.72**  (1.29-2.28) |
| A serious injury or illness of close family members | 13.7 | 1.26  (0.84-1.88) | 1.27  (0.97-1.67) |
| Conflict between parents | 11.8 | **2.41**  (1.63-3.56) | **1.94**  (1.45-2.60) |
| The death of a close friend | 5.6 | 0.67  (0.33-1.38) | 0.87  (0.56-1.34) |
| A serious injury or illness (participating child) | 5.1 | 1.58  (0.89-2.80) | 1.25  (0.83-1.87) |
| Mental disorder in the immediate family | 3.4 | **3.40**  (1.85-6.28) | **2.10**  (1.28-3.47) |
| Drug-taking or alcoholism in the immediate family | 3.2 | **2.72**  (1.33-5.59) | **1.82**  (1.03-3.23) |
| The death of a parent | 2.4 | **2.75**  (1.20-6.32) | 0.88  (0.41-1.90) |
| Another unspecified event | 1.7 | 1.67  (0.58-4.85) | 1.79  (0.89-3.63) |
| A stay in foster home or residential care | 1.2 | 2.20  (0.65-7.54) | 0.53  (0.16-1.74) |
| A parent in prison | 0.9 | 2.74  (0.81-9.23) | 0.96  (0.22-4.15) |

Note: OR: Odds Ratio. Emboldened metrics denote significant differences (p <.05).

Supplementary Table 4. Counterfactual risk differences of the total, direct and indirect relationship between Cumulative Childhood Stress and late adolescent psychopathology.

| Mediator Variables | Single Variable Counterfactual mediation ^a^ | | | | Multivariate Non-Linear Probability Path-Decomposition | |
| --- | --- | --- | --- | --- | --- | --- |
|  | Total Effect  *RD* | Average Mediation  *RD* | Average Direct Effect  *RD* | Percentage of Average Mediated Effect | Average Mediation  *OR* | Percentage of the Average Mediation |
| Childhood Adversity and Adolescent Internalizing Problems | | | | |  |  |
| Parent Child Conflict | 1.6  (0.7-2.4) | 0.6  (0.3-0.7) | 1.0  (0.1-1.8) | **36.3** | 1.05  (1.02-1.07) | **33.6** |
| Parent Child Positive | 1.6  (0.8-2.4) | 0.01  (0.01-0.1) | 1.6  (0.7-2.3) | **5.6** | 0.99  (0.99-1.01) | 1.25 |
| Self-Concept | 1.5  (0.6-2.3) | 0.4  (0.2-0.5) | 1.1  (0.3-2.0) | **23.2** | 1.03  (1.01-1.04) | **18.87** |
| Physical Activity | 1.6  (0.7-2.4) | 0.01  (0.01-0.2) | 1.5  (0.7-2.3) | **7.9** | 1.01  (1.00-1.01) | **5.16** |
| Childhood Adversity and Adolescent Externalizing Problems | | | | |  |  |
| Parent Child Conflict | 1.2  (0.6-1.7) | 0.5  (0.3-0.7) | 0.7  (0.1-1.2) | **39.4** | 1.10  (1.07-1.14) | **37.4** |
| Parent Child Positive | 1.3  (0.8-1.8) | <0.1  (<0.1-0.1) | 1.3  (0.8-1.7) | **4.7** | 0.99  (0.98-1.00) | 2.0 |
| Self-Concept | 1.3  (0.8-1.8) | <0.1  (<0.1-0.2) | 1.3  (0.8-1.7) | **6.4** | 1.01  (0.99-1.02) | 3.9 |
| Physical Activity | 1.3  (0.9-1.8) | <0.1  (-0.1-0.1) | 1.3  (0.9-1.8) | 2.0 | - | - |

Note: RD: Risk Difference, OR: Odds Ratio. Emboldened metrics denote significant differences (p <.05). All results were adjusted for Gender, Income, PCG highest level of education, Urbanicity, Nationality.

**Supplementary Analysis 2. Item response theory approach.**

**Item response theory.** This model was observed to be preferable to a 1-parameter model (see supplementary Table 5 below). The 2pl-IRT model is based on two item parameters difficulty and discrimination. Difficulty is the point on the trait scale at the median probability of endorsement. Less frequently endorsed ideas are shifted to the right on the latent trait. In this context, items that are less frequently endorsed have a difficulty that would indicate a higher levels of the adversity trait. Discrimination determines the probability of endorsement given similar levels of the latent trait. This parameter is based on the slope of the item characteristic curve and it describes how well an item can distinguish between individuals at different levels of the latent trait. In this context, higher discriminatory items would have greater ability to distinguish those who experienced adversity from those who did not at a given level of latent adversity trait. The prevalence discrimination value and difficulty of each adversity item can be found below in supplementary Table 6. We used this 2pl-IRT model to generate a latent adversity score for each participant (Mean: -0.08; SD: 0.63; Range: -0.63 – 3.78).

**Exposure-Outcome Relationship.** Using logistic regression, we examined the relationship between the predicted adversity latent trait score and psychopathology after adjusting for the same confounders as in the primary analysis in the main text. Childhood adversity was significant associated with an increased risk of both internalising (OR:1.41 95%ileCI: 1.21-1.64) and externalising problems: (OR:1.71 95%ileCI: 1.40-2.09).

**Exposure-Mediator Relationship.** We examined the relationship between the latent trait adversity score and all seven potential mediators using linear regression. Childhood adversity was significantly associated with parent-child conflict (β = 1.42, 95%ileCI: 1.00-1.84), parent-child positive (β =-0.32, 95%ileCI:-0.51 - --0.13), self-concept (β = -1.37, 95%ileCI:-1.93 - -0.81), computer use (β =0.17, 95%ileCI: 0.01 – 0.34) and physical activity (β = -0.12, 95%ileCI: -0.20 - -0.06). Childhood adversity was not significantly associated with peer trust (β =-0.32 95%ileCI: -0.79 – 0.16) and peer alienation (β =0.12, 95%ileCI:-0.14 – 0.38). All mediators were significantly associated with internalising and externalising problems (see Table 2 in the main text).

**Mediation Analysis.** The same statistical methods were was used as in the main text. The results are reported in supplementary Table 7. In spite of the differences in approaches to calculating our adversity measure with the main text (three or more minor stressors and/or one major stressor) and our a latent adversity measure, the results are consistent. Parent-childhood conflict accounts for roughly 30-40% of the relationship between childhood adversity and late adolescent psychopathology (both internalising and externalising problems). Self-concept and physical activity significantly mediated an additional proportion of the relationship between childhood adversity and late adolescent internalising problems which cumulatively accounted for the majority of this relationship.

Supplementary Table 5. Fit criteria and likelihood ratio test for the 1 parameter and the 2 parameter model.

| **Model** | **Log Likelihood** | **df** | **Chi Diff (p-value)** | **AIC** | **BIC** |
| --- | --- | --- | --- | --- | --- |
| 1 pl model | -30817.17 | 15 | 1450.12 | 61664.34 | 61770.18 |
| 2 pl model | -30092.11 | 28 | (>.001) | 60240.22 | 60437.78 |

Supplementary Table 6. The Prevalence, Discrimination value and Difficulty of each adversity item.

| **Childhood adversity types** | **%** | **Discrimination**  (95%CI) | **Difficulty**  (95%CI) |
| --- | --- | --- | --- |
| The death of a parent | 2.4 | **0.13**  (0.10-0.37) | **26.79**  (-18.46-72.04) |
| The death of a close family member | 43.2 | **-0.15**  (-0.22 - -0.08) | **-1.83**  (-2.78 - -0.88) |
| The death of a close friend | 5.6 | **0.17**  (0.02 - 0.33) | **15.98**  (2.17-29.80) |
| Divorce or separation of parents | 14.1 | **2.73**  (2.37-3.09) | **1.25**  (1.18-1.32) |
| Moving House | 41.4 | **0.81**  (0.71 - 0.91) | **0.48**  (0.39 - 0.56) |
| Moving Country | 9.6 | **0.66**  (0.53 - 0.78) | **3.66**  (3.03-4.28) |
| A stay in foster home or residential care | 1.2 | **1.26**  (0.96-1.57) | **4.10**  (3.35-4.85) |
| A serious injury or illness (participating child) | 5.1 | **0.44**  (0.28 - 0.60) | **6.82**  (4.53-9.11) |
| A serious injury or illness of close family members | 13.7 | **0.44**  (0.33 - 0.54) | **4.33**  (3.34-5.32) |
| Drug-taking or alcoholism in the immediate family | 3.2 | **1.70**  (1.45-1.95) | **2.69**  (2.45-2.93) |
| Mental disorder in the immediate family | 3.4 | **1.41**  (1.20-1.63) | **2.94**  (2.63-3.25) |
| Conflict between parents | 11.8 | **4.43**  (3.42-5.45) | **1.24**  (1.17-1.31) |
| A parent in prison | 0.9 | **1.83**  (1.43-2.22) | **3.40**  (2.95-3.85) |
| Another unspecified event | 1.7 | **0.67**  (0.41-0.92) | **6.39**  (4.17-8.61) |

Note: Emboldened values indicate statistical significance at p <.05.

Supplementary Table 7. Counterfactual risk differences of the total, direct and indirect relationship between latent trait childhood adversity and late adolescent psychopathology.

| Mediator Variables | Single Variable Counterfactual mediation ^a^ | | | | Multivariate Non-Linear Probability Path-Decomposition | |
| --- | --- | --- | --- | --- | --- | --- |
|  | Total Effect  *RD* | Average Mediation  *RD* | Average Direct Effect  *RD* | Percentage of Average Mediated Effect | Average Mediation  *OR* | Percentage of the Average Mediation |
| Childhood Adversity and Adolescent Internalizing Problems | | | | |  |  |
| Parent Child Conflict | 4.6  (2.3-7.0) | 1.5  (0.9-2.0) | 3.0  (0.8-5.4) | **32.5%** | 1.10  (1.06-1.14) | **29.13** |
| Parent Child Positive | 4.6  (2.3-7.0) | 0.03  (<0.1-0.4) | 4.3  (2.1-6.7) | **5.88%** | 0.99  (0.98-1.01) | 1.46 |
| Self-Concept | 4.3  (1.9-6.8) | 1.0  (0.6-1.4) | 3.3  (1.1-5.7) | **22.9%** | 1.06  (1.03-1.09) | **16.97** |
| Physical Activity | 4.7  (2.4-7.1) | 0.4  (0.1-0.7) | 4.3  (2.0-6.7) | **9.10%** | 1.01  (1.00-1.03) | **5.24** |
| Computer Use | 4.7  (2.4-7.1) | 0.2  (0.01-0.3) | 4.5  (2.2-6.9) | **3.32%** | 1.01  (0.99-1.01) | 1.52 |
| Childhood Adversity and Adolescent Externalizing Problems | | | | |  |  |
| Parent Child Conflict | 3.8  (2.0-6.0) | 1.5  (1.0-2.0) | 2.4  (0.6-4.4) | **38.01%** | 1.24  (1.16-1.31) | **40.23%** |
| Parent Child Positive | 4.3  (2.4-6.4) | 0.2  (<0.1-0.4) | 4.1  (2.2-6.2) | **5.44%** | 0.99  (0.97-1.00) | 2.64% |
| Self-Concept | 4.3  (2.3-6.5) | 0.3  (0.1-0.5) | 4.0  (2.0-6.2) | **7.25%** | 1.02  (0.99-1.05) | 4.29% |
| Physical Activity | 4.5  (2.5-6.8) | 0.1  (<-0.1-0.2) | 4.4  (2.5-6.7) | 2.38% | - | - |
| Computer Use | 4.5  (2.6-6.7) | <0.1  (<-0.1-0.2) | 4.5  (2.5-6.6) | 1.90% | - | - |

Note: RD: Risk Difference, OR: Odds Ratio. Emboldened metrics denote significant differences (p <.05). All results were adjusted for Gender, Income, PCG highest level of education, Urbanicity, Nationality.
